# Training Experimental Biologists in Bioinformatics

**DOI:** 10.1155/2012/672749

**Published:** 2012-01-31

**Authors:** Pedro Fernandes, Pooja Jain, Catarina Moita

**Affiliations:** ^1^Instituto Gulbenkian de Ciência, Rua da Quinta Grande, 6, 2780-156 Oeiras, Portugal; ^2^Centre for Integrative Systems Biology and Bioinformatics, Division of Molecular Biosciences, Imperial College London, London SW7 2AZ, UK; ^3^Unidade de Biologia Celular do Sistema Imunitário, Instituto de Medicina Molecular, Faculdade de Medicina, Universidade de Lisboa, 1649-028 Lisboa, Portugal

## Abstract

Bioinformatics, for its very nature, is devoted to a set of targets that constantly evolve. Training is probably the best response to the constant need for the acquisition of bioinformatics skills. It is interesting to assess the effects of training in the different sets of researchers that make use of it. While training bench experimentalists in the life sciences, we have observed instances of changes in their attitudes in research that, if well exploited, can have beneficial impacts in the dialogue with professional bioinformaticians and influence the conduction of the research itself.

## 1. Introduction

Bioinformatics uses biological information to study biological problems in a wide range of scales with the help of computer science methods. The adoption of Bioinformatics methodologies reaches areas that go from simple quantitative assessments that can help to solve a single focus problem, to projects that lead to the development of fully fledged workflows, employing a wide diversity of tools for addressing large-scale, data intensive analytical tasks. It is very difficult to build an exhaustive, categorized list of the multidisciplinary skills that are required.

The diversity of backgrounds and professional aims of the trainees, the constant evolution of Bioinformatics methods, and the heterogeneity of the data resources are the major sources of difficulty that a provider of quality training needs to identify and characterize.

Practical skills in Bioinformatics need to be acquired on top of existing knowledge. The first exposure to Bioinformatics methods has often been the result of participating in workshops, summer schools and training courses, collaborations and direct transfer of skills from colleagues, or just self-study.

Most people that use Bioinformatics professionally have graduate level studies in Biochemistry, Biology, Medicine, Biotechnology, and more rarely, in Mathematics, Computer Science, Chemistry, or Engineering. The need for cross-disciplinary training exists, naturally, as Bioinformatics resulted from the confluence of these disciplines.

## 2. The Origin of the Needs

It has become absolutely crucial for experimentalists to acquire at least basic skills to query, retrieve, and relate the biological information that is constantly accumulating in various databases. Otherwise, the risk of becoming “illiterate” and being outpaced in the respective field rises sharply. This pressure has very much revolutionized the experimentalists' attitude in research, increasing awareness about the data and information resources, aimed at identifying existing evidence and exploration of working hypothesis. Progressively, the role of literature is being shifted to more encompassing resources (literature plus data). Moreover, the extensive integration of information from different data resources has broadened the capacity to look at alternative interpretations and the need to generate new strategies to consider/discard new experiments.

As high-throughput experimentation is becoming routine, the need for Bioinformatics skills has increased at various levels. Handling biological information from these sources requires an additional set of nontrivial skills, appropriate for handling large amounts of information. The rapid evolution of the new sequencing platforms also makes it more difficult to deliver training in a stable way. However, it is clear that the availability of affordable sequence data will progressively move from laboratories and small core facilities to industrial facilities. Even when sequencing fragment size predictably increases, researchers will need to deal with data in many fragments with smaller overlap and less data reading reliability per fragment.

## 3. Grouped Typification of Trainees

Researchers that have left school with a background in either Biochemistry, Biology, Genetics, and so forth, or Mathematics, Physics, and Engineering, and have found value in Bioinformatics later in their careers, have common characteristics as candidate trainees that allow for a relatively easy characterization as a target group, in spite of their diversity. They typically seek training themes that are focused on specific techniques. They exhibit a preference for mono-thematic subjects and are much more concerned with problem-solving reliability, prediction accuracy, and quality control.

In the last two decades, the availability of various post-graduate courses in Computational Biology and Bioinformatics has been opening new options for students to access educational programs while still at the university. Nowadays, students can envisage undergraduate studies with Bioinformatics in mind. The skills that are provided in such courses are tailored to the educational objectives and are often missing the more practical, hands-on experience. They know more about the general approaches to problem solving than about the skills needed to create a data analysis pipeline in an industry. As such, students that follow these courses start off professionally from graduate school and constitute a second target category of recipients for training. What they look for is either a technological update (how to annotate a new genome from a nonmodel organism from scratch, e.g.,) or the specific skills to work on an area of research that they might be unfamiliar with (such as in Chemoinformatics in screening compounds for toxicity, e.g.).

There is a third category that consists of experimentalists in the life sciences. For such professionals, acquiring Bioinformatics skills at any level is a significant step towards embracing biological problem solving considering the information side in tight combination with wet lab experiments.

Regardless of the original background and the training method that is used, the acquisition of Bioinformatics skills has a professional impact. In a relatively small number of cases, it can cause dramatic changes in the professional profile, yielding professionalized bioinformaticians. Otherwise, there are milder effects that produce observable changes in research attitudes.

We are interested in discussing the mild effects of exposure to training in Bioinformatics, when a person's professional profile is not significantly changed, yet impacts are visible in research attitudes, as we will try to illustrate.

The motivation for addressing this subject came from informal conversations with former trainees, that spontaneously reported that they were not conducting their laboratory research in the same way after an intensive contact with Bioinformatics (in training courses).

## 4. Exposing Bench Experimentalists to Bioinformatics

Our universe of observation consists of people that enrolled in short Bioinformatics hands-on training courses of the GTPB programme (over 2000 participants) [1]. Participants in these courses provide feedback through questionnaires at the end of each course, aimed at making improvements in the training methods. This kind of feedback is also very useful to show the evolving needs of participants, immediately after exposure to training, but they cannot possibly reveal the long-term effects that require time to settle in, and more contact with experimental laboratory life. To further explore the effects of training, occasional follow-up contacts with course participants have allowed us to directly observe changes in research attitudes. Our observations are further extended by contacts with experimental biologists that never took our courses but participated in live debates on issues related to the roles and missions of Bioinformatics [2].

The vast majority of the audience in GTPB courses consists of experimental biologists that are looking for effective ways of using Bioinformatics in their research. In broad terms, these course participants expect to acquire practical skills that can be used with independence, in combination with laboratory bench work. These expectations, when met, should allow them to perform their jobs better. But, actually quite often, they realize and report back that this exposure to training has brought them to a new attitude towards finding added value in the uses of biological information and enhanced ways of extracting knowledge from it.

One of the above mentioned follow-up contacts was conducted recently by surveying former course participants using e-mail. A set of questions was sent to 200 former participants, randomly chosen from a pool of 481 that attended GTPB courses since January 2010. All the responders (53) were inquired some time later than the course participation, allowing for the chance to apply the newly acquired skills. The aim was to gather some information on the real use of those skills after each course and, among other monitoring objectives, to detect possible changes in their research attitudes. Our sample consisted of 38 people that declared that they are wet-lab biologists, and the results are displayed in Table 1.

The results of the survey show

- a quite widespread perception of a positive impact of training in each participant's work;
- a high level of self-consciousness regarding a change in research attitude;
- a relatively high number of publications in less than two years;
- enrollment in more training events (further interest) being generated.

Naturally, the reasons for an affirmative answer in Q2 are diverse. In Table 2 several of these reasons are listed for comparison.

This collection of answers is useful in revealing how different viewpoints are used to justify change in attitudes. The simplest ones are the direct awareness of the ability to try using the newly acquired methods without constraints (as in A6 or A9). More elaborate answers reveal a deeper change, with a deeper consequence in the way of thinking about connected problems (A2) and Biology in general (A7).

This suggests that such changes in research attitudes, not being deliberately imposed, may result from the exposure to the methodologies, often shaped by the way in which the instructors decide to address the subjects. Usually, in GTPB, the instructors are asked not to explore the subjects very deeply, using the lectures to convey a conceptual framework in which the skills may fit. In many cases this is just what is needed to make the attendees understand how adequate the methods are, and how limited they can be in usage, before jumping into a hands-on exercise. Knowing who the attendees are and giving them a small amount of individual attention, the instructors can use the course as a way to help the attendees in finding appropriate changes in their research attitudes.

In Bioinformatics, these changes happen easily. With the exposure to abundant and diverse information resources, training course participants can often realize the importance of observing a phenomenon from different viewpoints and at different levels. Similarly, while switching between reductionist approaches and network or systems level ones, trained Bioinformatics practitioners can more easily find ways of deciding which is best at each stage of a research job. Another aspect concerns the use of techniques, as it is relatively easy to ask questions on hypothesis generation and testing, that the participants can perceive as ways to place the results of their experiments in full perspective, compare simulated results with experimental observations, and evaluate the predictive power and extrapolation of *in silico* methodologies [3].

## 5. Concluding Remarks

Bioinformatics training can promote attitude changes in some of the core aspects of scientific research. Changes in research attitudes, as perceived by trainees and trainers, can occur in specific aspects of laboratory practice, influencing the way in which wet-lab experiments are conducted. They can also occur in less-focused situations. For example, some of these former trainees have discovered the advantages of comparing laboratory results with those produced by predictive simulations using models. These nonrare occurrences, if studied in more depth, can provide clues about effective ways of extending the usefulness of Bioinformatics training.

One possible way would be to systematically stimulate course participants to find this added value, when it is somewhat hidden. This is an additional role that a good trainer usually likes to take on his shoulders. For example, the concept of “validation” can be explored in instances where it implies checking results obtained with Bioinformatics with wet laboratory experiments, but it can also be explored by finding quantifiable agreement in results obtained with independent Bioinformatics methods on the same or on heterogeneous data resources. The trainer can use the occasion to show how to evaluate confidence in results, sensitivity, robustness, and so forth. The trainer can also show how to ensure applicability of a method in a rigorous way. In broad terms, these are concepts that allow the trainee to see counterparts in laboratory work that can be extremely valuable for steering it.

Another way would be to influence the design of training courses and their provision. The careful combination of problem solving approaches and Bioinformatics techniques can lead to well-supported hypothesis formulation and testing and contribute to research as a controlled quality source of methods for evidence-based prediction. Contact with training that is designed for displaying this kind of goal seeking attitude in mind, is likely to produce more positive results. Unlike most formal training in traditional Computer Science subjects, training in Bioinformatics is not usually set up as a sequence of interdependent modular courses, following a predetermined path [4]. However, it is possible to identify areas of interest in Biology where, considering the characteristics of the trainees, the available opportunities and the potential benefits, and sets of independent training courses can be chosen. Providing this kind of guidance is bound to prove extremely useful for biologists seeking such opportunities and could help to enhance their impact. To make it possible, the biggest obstacle is still in the restricted availability of training courses, held regularly and with tractable contents [5].

In the most common public perception, Bioinformatics training is limited to the provision of skills as the need arises. That conceals the necessity for its insertion in more encompassing research activities such as the overall buildup of research capabilities, and quite a large part of its potential usefulness. Our view is that experimental (bench and field) biologists increasingly need access to well-designed training in Bioinformatics, in ways that clearly connect their professional activity at the bench with other aspects of research that are much more driven by information-based methodologies. Biologists that are equipped with Bioinformatics skills feel much more able to take the most of interacting with bioinformaticians, who would not easily take the inverse path and opt for working at the bench, for example. In this sense, training biologists in Bioinformatics, rather than subtracting relevant roles from the hands of bioinformaticians, is an ingenious way of enhancing the interplay, while opening chances of promoting the best interdisciplinary practices. If the attitudes towards research change, some extra value can be added. Stimulating trainees for this to happen is a matter of being able to discover these opportunities and consciously take the most from them.

## Figures and Tables

**Table 1:** Results of the survey on former course participants 2010-2011 (*n* = 38).

Question	Yes	No	Unclear answer
(Q1) Did the skills learnt here have a positive impact in your work?	34	2	2
(Q2) Did your research attitude change towards seeking added value by using biological information and Bioinformatics methods?	29	7	2
(Q3) Can you reference a publication in which you have used what you learnt here?	7	18	13
(Q4) Did you enroll in more Bioinformatics training courses?	11	24	3

**Table 2:** Sample responses regarding the reasons for the observed change (“redundant” answers have been omitted for clarity).

A1	“I have (acquired) a new perspective about the biological questions involved in my research lines that previously I hadn't. I have perceived the importance and the utilities of managing biological databases in order to approach my research, mainly with regard to gene functional annotation.”

A2	“(…) by making me better understand published work and knowing (about) additional Bioinformatics tools at my disposition.”

A3	“Yes and no, I already knew that bioinformatics methods could add value, but your course showed me the tools available to put this into practice.”

A4	“I have done the MEPA11 course that led me to think much more about what I'm doing evolution-wise, and how some of the stuff I'm doing may be analyzed, using some of the tools I have learned in the course.”

A5	“The attendance of the Bioinformatics training courses gave me the skills to begin a new line of research, concerning molecular evolution.”

A6	“Yes, I'm currently analyzing data and I'm trying to incorporate the knowledge obtained in GTPB Course as an added value to the previously projected statistical analysis.”

A7	“Yes, Bioinformatics tools and methods brought to the “traditional” biology means of analyzing and simulating biological data (capacity of analyzing large amounts of data), and obtain a couple of possible results that can be later verified in a laboratory.”

A8	“Yes, I now try to take more advantage from the Bioinformatics tools for data exploration but also for prediction or statistical validation of the data sets.”

A9	“Yes. Through a better awareness of available methods and tools and how they may help answer questions in my research line.”
